# Surface emergence and persistence of MHC class I free heavy chains

**DOI:** 10.1016/j.jbc.2025.110799

**Published:** 2025-10-09

**Authors:** Fernando M. Ruggiero, François-Xavier Mauvais, Ursula Wellbrock, Peter M. van Endert, Sebastian Springer

**Affiliations:** 1 School of Science, Constructor University, Bremen, Germany; 2 Université Paris Cité, INSERM, CNRS, Institut Necker Enfants Malades, Paris, France; 3 Service de Physiologie - Explorations Fonctionnelles Pédiatriques, AP-HP, Hôpital Universitaire Robert Debré, Paris, France; 4 Service Immunologie Biologique, AP-HP, Hôpital Universitaire Necker - Enfants Malades, Paris, France

**Keywords:** major histocompatibility complex (MHC), membrane recycling, trafficking, kinetics, plasma membrane, beta2-microglobulin dissociation, cell surface retention, free heavy chains (fH), *in silico* modeling, cross-presentation

## Abstract

Major histocompatibility complex (MHC) class I heavy chains associate with the light chain beta-2 microglobulin (β_2_m) to present antigenic peptides to cytotoxic T cells. Upon dissociation of both peptide and β_2_m, class I free heavy chains (fH) are generated. While the presence of the fH at the cell surface has been reported, their biogenesis, trafficking, and lifetime are insufficiently defined, and their biological functions are largely unknown. Here, we show that class I fH arise *via* β_2_m dissociation from peptide-free, β_2_m-bound class I molecules that are located at the plasma membrane or recycled from intracellular compartments. Using conformation-specific antibodies, peptide rescue assays, brefeldin A blockade, and *in silico* modeling, we elucidate the short- and long-term kinetics of generation and disappearance of plasma membrane-associated fH. We demonstrate that fH accumulate at the cell surface within minutes and persist for several hours, with rates of endocytic removal and recycling varying substantially between the analyzed allotypes. Notably, a fast-recycling pool of human leukocyte antigen (HLA) class I fH exists in HeLa cells, whereas murine H-2L^d^ and H-2D^b^ recycle more slowly. These findings elucidate both the early origins of cell surface fH and the kinetics governing their production and disappearance, and they highlight their prolonged surface residency as a potential determinant of novel biological roles.

The presentation of peptides to cytotoxic T lymphocytes by major histocompatibility complex (MHC) class I molecules is central to the adaptive immune response against intracellular pathogens and tumors (1). MHC class I molecules consist of a non-covalent complex of the polymorphic class I heavy chain (H), the monomorphic light chain beta-2 microglobulin (β_2_m, or β), and a peptide (P). After proper folding and peptide loading, the fully assembled HβP complex exits the endoplasmic reticulum (ER) and traffics to the cell surface (2, 3). Its removal from the plasma membrane is determined by dynamic cycles of endocytosis and recycling and by delivery to lysosomes for degradation (4, 5, 6).

While the stability of MHC class I molecules depends on the binding of β_2_m and peptide to the heavy chain (7, 8, 9), additional peptide-free forms have been detected at the cell surface under physiological and pathological conditions and in various cell lines. These include peptide-receptive (‘empty’) Hβ dimers and the β_2_m-free heavy chain, referred to as the free heavy chain (fH). Surface Hβ can originate by peptide dissociation from the HβP complex either at the cell surface or else in endocytic compartments, followed by recycling (10, 11). It is also known that some low-affinity peptides that are bound in the ER dissociate during the exocytic trafficking or shortly after arrival at the cell surface (12), further contributing to the surface Hβ pool.

The presence of class I fH at the plasma membrane is well established (13, 14, 15, 16, 17, 18, 19, 20, 21, 22, 23, 24, 25, 26, 27, 28, 29, 30, 31, 32, 33, 34, 35, 36, 37, 38, 39), and multiple mechanisms may contribute to its generation. The appearance of fH on the cell surface was proposed to require the transit of class I molecules through the endocytic compartments (22, 23, 40) followed by recycling of the fH (41). However, other studies reported rapid lysosomal degradation of endosomal fH, suggesting limited opportunity for recycling (8, 42). Other reports showed that fH arises directly at the cell surface after peptide and β_2_m dissociation *in situ* (8, 13, 14, 25, 26, 27, 28). Class I fH of some allotypes was also detected at the surface of β_2_m-negative cells, or when β_2_m was retained in the ER (32, 33, 34, 35, 36).

Whatever the mechanism involved, the fH remained at the plasma membrane for extended periods (13, 14). Still, these older studies assessed fH levels at hour-long intervals, leaving the short-term dynamics of these species poorly resolved.

In the present study, we sought to improve temporal resolution by examining fH emergence at shorter time intervals, on the scale of minutes, thereby allowing us to better distinguish between early and late events following the fH appearance at the surface. To this end, we used conformation-specific antibodies, the anterograde trafficking inhibitor brefeldin A, and high-affinity peptide supplementation to dissect the origin and fate of surface-localized MHC class I fH.

We propose that class I fH are rapidly generated at the surface of TAP-deficient and TAP-inhibited cells through β_2_m dissociation from Hβ molecules located at the plasma membrane and from Hβ that recycle back to the cell surface. The dissociation of β_2_m proceeds fast and leads to the accumulation of surface fH within minutes. Once formed, these fH remain stably associated with the plasma membrane for extended periods. Remarkably, their persistence varies among different class I allotypes, suggesting that the rate of fH removal is not uniform and may reflect intrinsic stability differences or differential engagement by clearance mechanisms.

Our findings advance the understanding of fH biogenesis by capturing both their rapid appearance and their variable rate of removal among allotypes, raising the possibility that this variation may have functional consequences. This challenges the view that fH are merely inert byproducts of class I degradation. Notably, the fH can associate into clusters at the plasma membrane (43). The extent to which different MHC class I allotypes form or retain surface clusters, the mechanisms underlying these processes, and their potential biological functions remain unresolved questions and merit further investigation.

## Results

### Temperature-dependent cell surface accumulation of “empty” MHC class I heterodimers (Hβ) on TAP-deficient and TAP-inhibited cells

Three forms of class I molecules are present at the cell surface: fH (free heavy chains), Hβ (fH plus β_2_m), and HβP (Hβ plus peptide). To simplify our analysis of the generation and fate of fH at the cell surface, we used cells that lack a functional transporter associated with antigen processing (TAP), which typically show reduced peptide translocation into the ER. Although some MHC class I allotypes exhibit partial TAP independence, TAP-deficient cells overall display reduced peptide loading, resulting in very low levels of HβP molecules at the surface (44, 45). Early works showed that Hβ found at the surface of TAP-deficient cells rapidly dissociates into its individual components at 37 °C. At 25 °C; however, they are much more resistant to dissociation (46, 47). At this temperature, they accumulate at the cell surface, where they can bind exogenous peptides (46, 47). This low temperature effect is more pronounced with murine than with human class I molecules (48). Following 25 °C incubation, a shift to 37 °C triggers the generation of fH (46, 47). Thus, TAP-deficient cells constitute a suitable working model to study fH dynamics.

**Figure 1:**
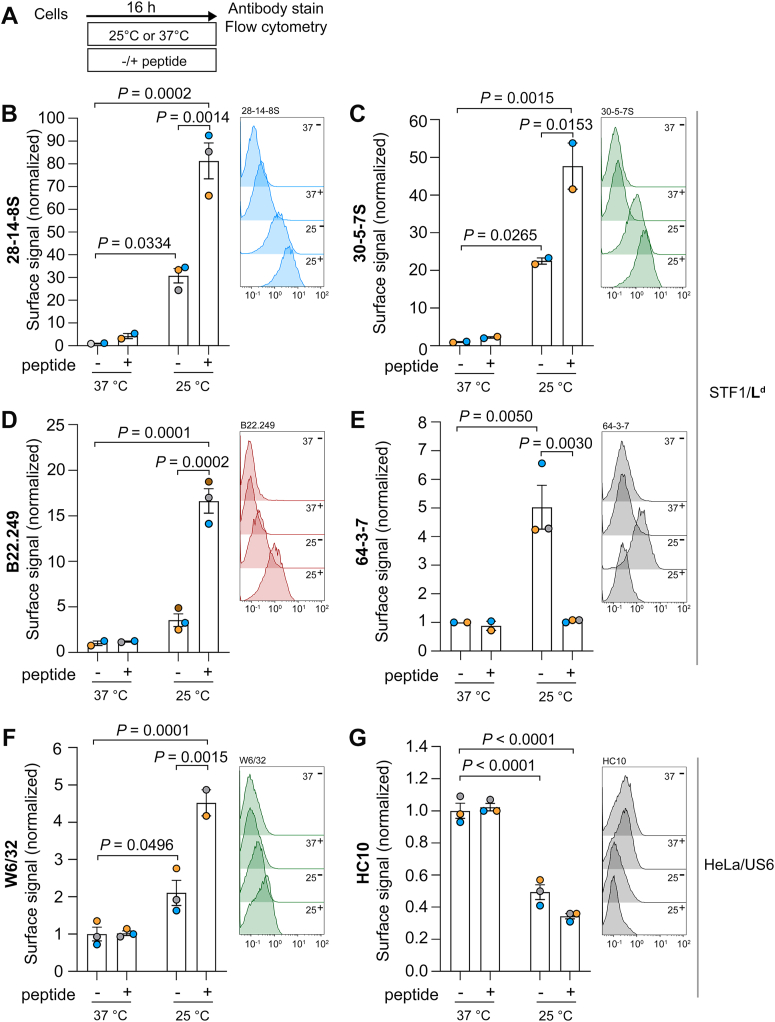
**Accumulation of peptide-free, β_2_m-bound (Hβ) MHC class I molecules and free heavy chains (fH) at the cell surface.***A*, TAP-deficient STF1 cells expressing H-2L^d^ (STF1/L^d^) and TAP-inhibited HeLa cells (HeLa/US6) were incubated overnight at 37 °C or 25 °C in the absence or presence of 10 μM of peptides. *B-G*, cells were processed for flow cytometry using the antibodies indicated in each panel. An overview of the class I species recognized by each mAb is provided in Fig. S1*A*. The graphs show the fluorescence intensity relative to the signal at 37 °C without peptides (control). The presence (+) or absence (−) of peptide and the incubation temperature (37 or 25 °C) are indicated below. Data are presented as mean ± S.E.M. Statistical significance analysis was assessed using two-way ANOVA followed by Tukey's multiple comparison test. Only significant changes are indicated with their corresponding *p* values. Each data point represents one independent biological experiment (mean of technical replicates). Two to three independent experiments were performed per condition. Representative flow cytometry histograms are shown alongside each plot, with the x-axis showing the absolute fluorescence intensity values. The incubation temperature (37 or 25 °C) and presence (+) or absence (−) of peptide are indicated next to each tracing.

To investigate the relative levels of Hβ and fH at the cell surface, we used human TAP-deficient STF1 cells (49) stably expressing the murine class I molecule H-2L^d^ (in the following, L^d^). STF1/L^d^ cells were incubated overnight at 37 °C or at 25 °C in the presence or absence of 10 μM of cognate peptide. Following incubation, surface levels of the different L^d^ forms (L^d^-HβP, L^d^-Hβ, or L^d^-fH) were determined by flow cytometry (Fig. 1*A*). To distinguish between these species, we employed a panel of conformation-specific monoclonal antibodies (mAbs). A summary of their specificities is provided in Fig. S1*A*, and the key mAbs used in this study are also depicted in the schematic model shown in Figure 6.

**Figure 2:**
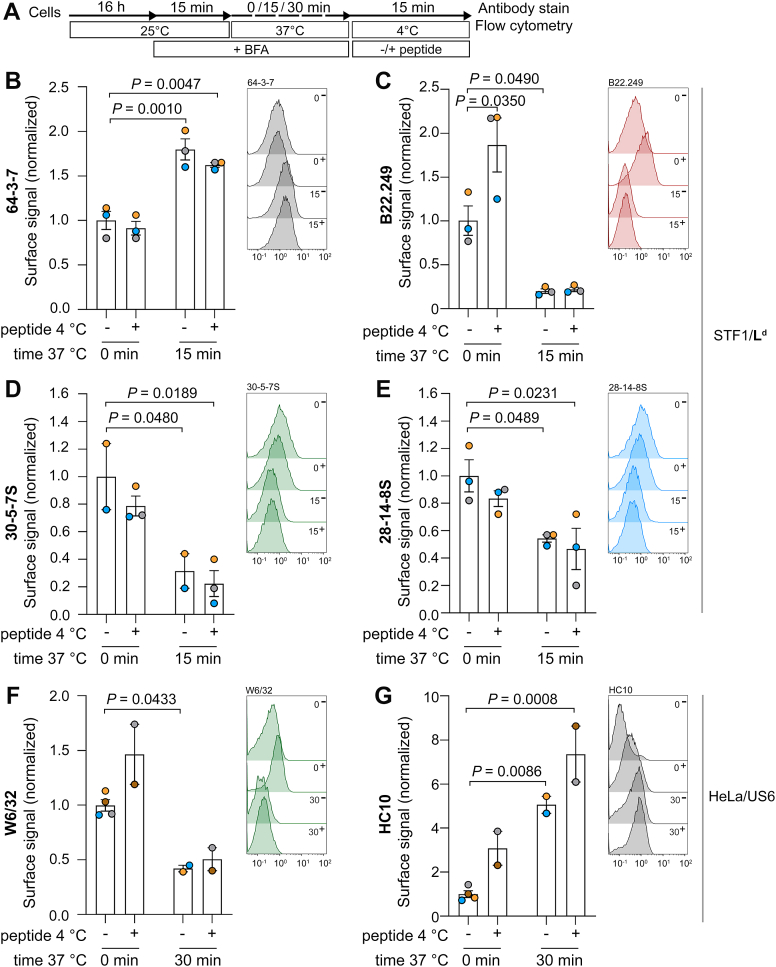
**The monoclonal antibodies 64-3-7 and HC10 bind cell surface L^d^-fH and HLA-fH.***A*, cells were incubated overnight at 25 °C, then transferred to DMEM containing 10 μg/ml Brefeldin A (BFA) for 15 min at 25 °C. Subsequently, the medium was replaced with pre-warmed DMEM containing 10 μg/ml BFA, and cells were incubated at 37 °C for 15 min (STF1/L^d^) or 30 min (HeLa/US6). Control samples were kept at 25 °C (0 min at 37 °C). After that, cells were harvested and incubated in the presence (+) or absence (−) of peptides at 4 °C for 15 min. *B–G*, surface levels of class I molecules were analyzed by flow cytometry using the antibodies indicated in each panel. An overview of the class I species recognized by each mAb is provided in Fig. S1*A*. The graphs show the fluorescence intensity relative to control (0 min of incubation at 37 °C, no peptide). The time that cells were incubated at 37 °C (0, 15 or 30 min), and the presence (+) or absence (−) of peptide during incubation at 4 °C are indicated below. Data are presented as mean ± S.E.M. Statistical significance analysis was assessed using two-way ANOVA followed by Tukey's multiple comparison test. The *p* values are indicated for those conditions statistically associated with a significant change in signal. Each data point represents one independent biological experiment (mean of technical replicates). Two to four independent experiments were performed per condition. Representative flow cytometry histograms are shown alongside each plot, with the x-axis showing the absolute fluorescence intensity values. The time that cells were incubated at 37 °C (0, 15 or 30 min) and the presence (+) or absence (−) of peptide during the incubation at 4 °C are indicated next to each tracing.

**Figure 3:**
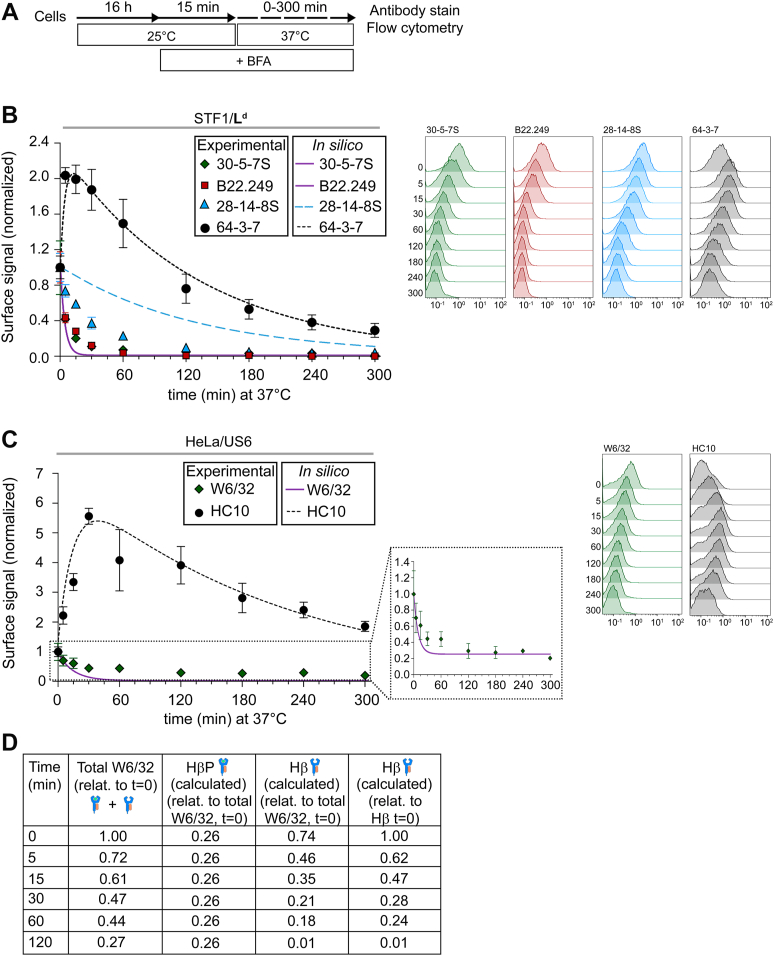
**Mouse and human class I free heavy chains (fH) emerge at the cell surface and remain plasma membrane associated for extended periods.***A*, cells were incubated overnight at 25 °C to accumulate Hβ and fH at the plasma membrane, followed by 15 min at 25 °C in DMEM containing 10 μg/ml brefeldin A (BFA). The medium was then replaced by 37 °C pre-warmed DMEM containing 10 μg/ml BFA, and cells were incubated at 37 °C for the indicated times. *B*-*C*. Surface levels of class I molecules were monitored by flow cytometry using the indicated antibodies in STF1/L^d^ (*B*) and HeLa/US6 cells (*C*). An overview of the class I species recognized by each mAb is provided in Fig. S1*A*. The plots show the fluorescence intensity for each mAb, at each time point, and relative to the corresponding control (0 min of incubation at 37 °C). Data are presented as mean ± S.E.M. *In silico* predictions are overlaid as follows: long-dashed curves for 28-14-8S (*B*); solid line for B22.249 and 30-5-7S (*B*) and W6/32 (*C*); short-dashed curves for 64-3-7 (*B*) and HC10 (*C*). A rescaled panel shows W6/32 decay curve highlighting the full extent of signal loss over time (*C*). At least three independent experiments per condition were performed. Representative flow cytometry histograms are shown next to each graph, with the x-axis showing the absolute fluorescence intensity values. The time that cells were incubated at 37 °C (minutes) is indicated to the left of the tracings. *D*, total measured W6/32 levels (HβP + Hβ) and the calculated fractions for each of the two species recognized by this antibody. All values are relative to the total measured W6/32 surface signal at time zero (0 min of incubation at 37 °C).

**Figure 4:**
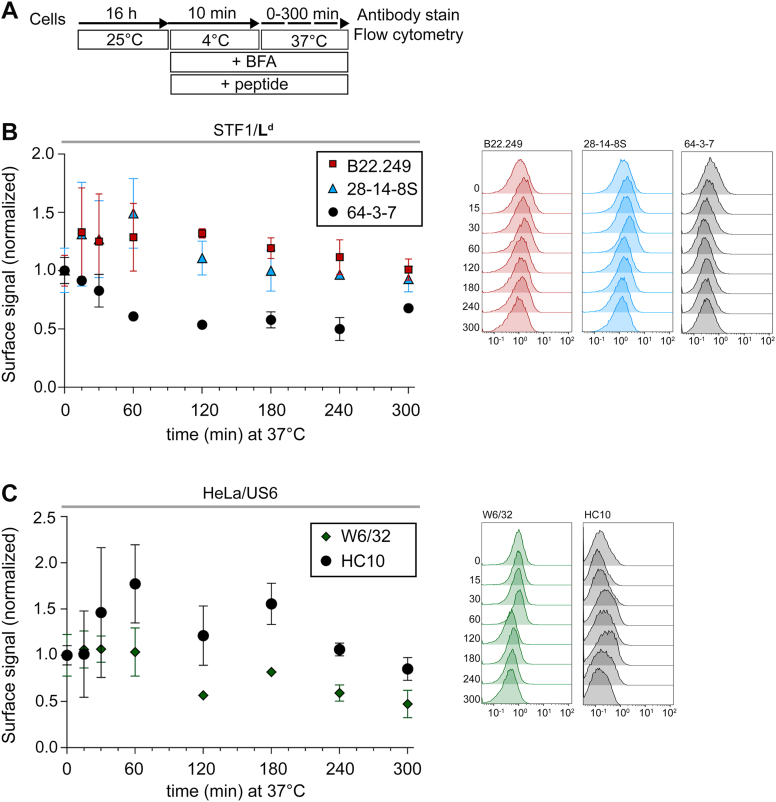
**Cell surface emergence of L^d^-fH is abolished, whereas HLA-fH is reduced by incubation with high-affinity peptides before and during the 25** **→** **37 °C temperature shift.***A*, cells were incubated overnight at 25 °C, followed by 10 min at 4 °C in DMEM containing 10 μM peptide and 10 μg/ml brefeldin A (BFA). The medium was then replaced with 37 °C pre-warmed DMEM containing 10 μM peptide and 10 μg/ml BFA, and cells were incubated at 37 °C for the indicated times. *B*-*C*. Surface levels of class I molecules were measured in three independent experiments by flow cytometry using the indicated monoclonal antibodies in STF1/L^d^ (*B*) and HeLa/US6 cells (*C*). An overview of the class I species recognized by each mAb is provided in Fig. S1*A*. The plots show the fluorescence intensity for each mAb, at each time point, and relative to the corresponding control (0 min of incubation at 37 °C). Data are presented as mean ± S.E.M. Representative flow cytometry histograms are shown next to each graph, with the x-axis showing the absolute fluorescence intensity values. The time that cells were incubated at 37 °C (minutes) is indicated to the left of the tracings.

**Figure 5:**
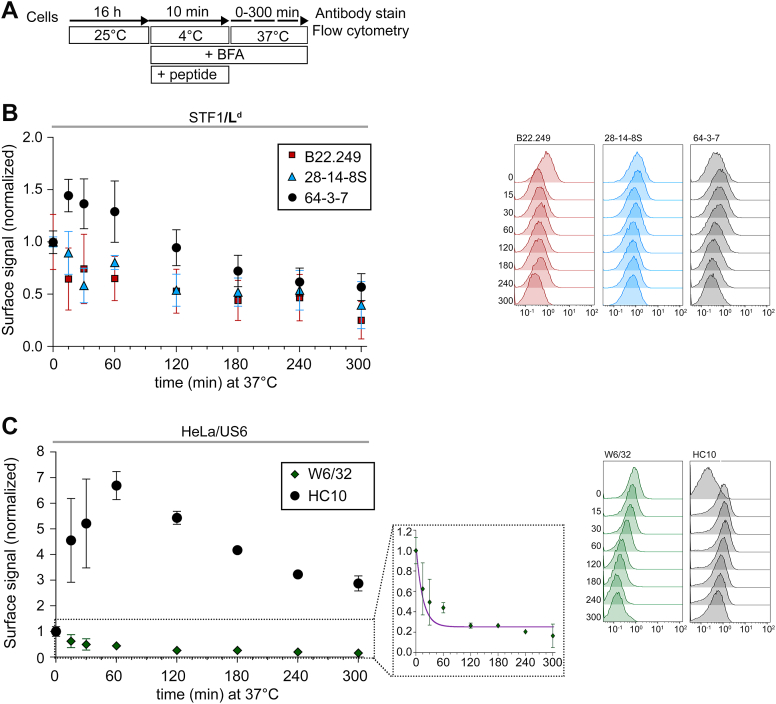
**A recycling Hβ pool contributes to the generation of surface L^d^-fH and HLA-fH.***A*. Cells were incubated overnight at 25 °C, then transferred to 4 °C for 10 min in DMEM containing 10 μM peptide and 10 μg/ml brefeldin A (BFA). After washing, the medium was replaced with 37 °C pre-warmed DMEM containing 10 μg/ml BFA, and cells were incubated at 37 °C for the indicated times. *B*-*C*. Surface levels of class I molecules were assessed by flow cytometry using the indicated monoclonal antibodies on STF1/L^d^ (*B*) and HeLa/US6 cells (*C*). An overview of the class I species recognized by each mAb is provided in Fig. S1*A*. The plots show the fluorescence intensity for each mAb, at each time point, and relative to the corresponding control (0 min of incubation at 37 °C). Data are presented as mean ± S.E.M. A rescaled panel shows W6/32 decay curve highlighting the full extent of signal loss over time (*C*). At least three and two independent experiments were conducted for STF1/L^d^ and HeLa/US6 cells respectively. Representative flow cytometry histograms are shown next to each graph, with the x-axis showing the absolute fluorescence intensity values. The time that cells were incubated at 37 °C (minutes) is indicated to the left of the tracings.

MAb 28-14-8S binds to the L^d^ α_3_ domain and therefore recognizes all L^d^ species (Hβ, HβP, and fH) (50, 51, 52, 53); 30-5-7S binds to the α_2_ domain of conformed L^d^ (Hβ and HβP) (15, 16, 54), and its binding is not influenced by the identity of the bound peptide (55); B22.249 binds to the α_1_ domain of conformed L^d^ (Hβ and HβP), and its recognition is modulated by the bound peptide, with optimal detection occurring when the ligand contains a proline at position 2 (55). Finally, 64-3-7 binds to the α_1_ domain of L^d^ and recognizes a form that was initially termed Ld^alt^. This form of L^d^ was originally described as an “open conformer” that is not bound to peptide nor β_2_m (15). However, a proportion of Ld^alt^ was no longer immunoprecipitated after peptide treatment of cell lysates, which led the authors to suggest that at least a fraction of the 64-3-7-positive L^d^ molecules can bind peptide (15). Later reports from the same group suggested that 64-3-7-positive Ld^alt^ molecules were “weakly” bound to β_2_m and can bind peptides in cell lysates (13, 16). However, 64-3-7-positive molecules detected at the cell surface behaved differently: they were refractory to peptide binding and were characterized as an unfolded form of L^d^ (13, 16). Furthermore, 64-3-7-positive Ld^alt^ molecules were also observed at the surface of β_2_m-negative cells (13). Thus, it is unclear what form of L^d^ is detected by 64-3-7 in cell lysates, but prior evidence suggests that at the cell surface, it recognizes L^d^-fH.

The epitope recognized by 64-3-7 has been structurally defined in complex with a short L^d^-derived peptide (56), but a detailed characterization of the full-length L^d^ form bound by 64-3-7 mAb at the cell surface (whether L^d^-Hβ or L^d^-fH) has remained incomplete. Since both species may differ in overall conformation, and thus, in the availability of the epitope to the antibody, we aimed to identify the L^d^ species recognized by 64-3-7 at the plasma membrane under our experimental conditions.

Incubation of STF1/L^d^ at 25 °C significantly increased the surface signal of mAbs 28-14-8S and 30-5-7S (Fig. 1, *B* and *C*). These results corroborate previous findings that low temperatures prevent β_2_m dissociation (46, 47). The signals of these mAbs showed even stronger increases when the peptide was added overnight at 25 °C, suggesting that surface Hβ had been converted to HβP (Fig. 1, *B*–*D*) since peptide binding stabilizes class I molecules, increasing β_2_m affinity for the heavy chain (46, 47). The 64-3-7 signal increased in STF1/L^d^ cells incubated overnight at 25 °C in the absence of peptide (Fig. 1*E*). But when the peptide was added during the overnight incubation at 25 °C, 64-3-7 labeling was considerably lower (Fig. 1*E*), indicating that the peptide diminishes the number of 64-3-7-positive forms at the cell surface. Therefore, 64-3-7 binds either to Hβ or to fH or to both, but not to HβP (15, 16).

We next asked whether HLA molecules behave similarly. STF1 cells express only very low levels of endogenous class I at the surface. Therefore, we used HeLa cells (naturally expressing HLA-A∗68:02, HLA-A∗19:03, HLA-B∗15:03 and HLA-C∗12:03) (57) stably transduced with the TAP inhibitor US6 from human cytomegalovirus (58, 59, 60). HeLa/US6 cells showed strongly reduced HLA-I surface levels compared to HeLa wild type (Fig. S1, *B* and *C*) for both mAb W6/32, which binds to all HLA-I allotypes as long as they are associated with β_2_m (*i.e.,* HβP and Hβ (61, 62)), and for mAb HC10, which primarily binds to HLA-fH (63) that contain the ^57^PxxWDR^62^ motif (64)) (Fig. S1*A*). Notably, HLA-A∗68:02, HLA-B∗15:03 and HLA-C∗12:03 carry this motif and may therefore contribute to the HC10 signal observed. Incubation of HeLa/US6 overnight at 25 °C increased the levels of HLA-Hβ (Fig. 1*F*), just like the rise in L^d^-Hβ observed in STF1/L^d^ cells (Fig. 1, *B* and *C*). A further significant increase in W6/32-positive molecules was observed after overnight incubation with high-affinity peptides at 25 °C, whereas the effect was abolished at 37 °C (Fig. 1*F*). These results show that, as with L^d^, peptide-receptive HLA-Hβ accumulate at the cell surface at 25 °C but not, or only to a small extent, at 37 °C.

Compared to the overnight incubation at 37 °C, incubation of HeLa/US6 overnight at 25 °C resulted in a 50% decrease in the levels of HC10-positive molecules (Fig. 1*G*). Overnight addition of exogenous peptide did not significantly decrease HC10-reactive class I molecules (Fig. 1*G*). These results show that, contrary to W6/32-positive molecules, HC10-reactive class I species expressed at the surface of HeLa cells preferentially accumulate at 37 °C and are peptide-insensitive.

Taken together, our data confirms that W6/32 and HC10 bind to different biochemical pools of HLA molecules. While low temperature promotes the accumulation of the W6/32-reactive species Hβ and HβP, the HC10-positive HLA molecules (HLA-fH) show a slight increase at physiological temperature.

### 64-3-7 and HC10 detect surface MHC class I free heavy chains

Results shown in Figure 1*E* suggest that mAb 64-3-7 binds either to surface L^d^-fH or L^d^-Hβ or both. Previous work by Mage and colleagues (56) provided structural insights into the 64-3-7 epitope, demonstrating that it binds a minimal sequence corresponding to L^d^ residues 46 to 53 (EPQAPWME). Crystallographic analysis of this peptide in complex with the Fv fragment of 64-3-7 revealed that binding requires exposure of W51, a residue buried in peptide-loaded L^d^ molecules but accessible in an altered conformation lacking peptide. Their modeling suggested that loss of peptide destabilizes the native 3_10_ helix in this region, facilitating the rearrangement necessary for 64-3-7 recognition.

The ^46^EPQAPWME^53^ sequence lies within a region of the L^d^ α_1_ domain adjacent, though not identical, to the HC10 epitope (^57^PxxWDR^62^) on the α_1_ domain of certain human class I allotypes (64). Like W51 in L^d^, the central W60 in the HC10 epitope is also buried in folded class I molecules and becomes exposed only upon unfolding. Therefore, while 64-3-7 and HC10 do not recognize the same epitope or class I molecules, both antibodies require significant unfolding of the α_1_ domain for binding. The structural perturbations described by Mage *et al.* (56) also disrupt the A and B pockets of the L^d^ peptide-binding groove, implying that the 64-3-7-positive conformation likely corresponds to a peptide-refractory state. Based on these similarities, we hypothesized that, under our experimental conditions, 64-3-7 binds to cell surface L^d^ that lacks peptide and β_2_m, *i.e.*, L^d^-fH, just like HC10 binds to HLA-fH.

To test which class I forms are bound by 64-3-7 and HC10 at the cell surface, we used a brefeldin A (BFA)/temperature upshift experiment. Since Hβ are thermolabile, a 25 °C to 37 °C temperature shift converts them into fH within minutes (11). We reasoned that, if 64-3-7 and HC10 recognize surface L^d^-fH and HLA-fH, an increase in the labeling with these mAbs should occur right after the temperature shift, whereas a decrease in signal should happen if they bind to L^d^-Hβ and HLA-Hβ. If both conformations are recognized equally, the signals will remain unchanged.

STF1/L^d^ were incubated overnight at 25 °C to accumulate surface L^d^-Hβ. Then, BFA was added, and cells were incubated for an additional 15 min at 25 °C (Fig. 2*A*). BFA blocks the anterograde transport but does not affect recycling from endosomes (65) nor the plasma membrane-to-lysosome trafficking (66). STF1/L^d^ cells were then incubated at 37 °C for 15 min to promote dissociation of β_2_m. Control samples (0 min at 37 °C) were left at 25 °C for the same time to preserve the association between class I heavy chain and β_2_m (Hβ). The cells were then transferred to 4 °C and incubated in the presence or absence of peptide (Fig. 2*A*). The low-temperature incubation halts endocytic and exocytic trafficking as well as β_2_m dissociation, ensuring that the cellular distribution of the class I forms is preserved for measurement. This is essential to guarantee that observed changes reflect surface events rather than intracellular redistribution.

Shortly after the 25 → 37 °C temperature shift, surface levels of 64-3-7-positive molecules increased (Fig. 2*B*, 15 min, no peptide), whereas B22.249, 30-5-7S and 28-14-8S signals decreased (Fig. 2, *C*–*E*, 15 min, no peptide). These results suggest that surface 64-3-7-positive molecules are L^d^-fH that arise from L^d^-Hβ upon β_2_m dissociation.

Interestingly, adding peptides on ice at the end of the experiment increased the surface signal for B22.249 (Fig. 2*C* and 0 min), which is unlikely to result from the generation of HβP from fH at the surface, since 30-5-7S remained constant (Fig. 2*D* and 0 min), and since cell surface fH do not bind or retain peptides in the absence of exogenous β_2_m (26, 67, 68, 69, 70). Nor does it suggest an increase in the total amount of L^d^, since the 28-14-8S signal remained constant (Figs. 2*E* and 0 min; compare 7; peptide), and since peptide incubation occurred at 4 °C and in the presence of BFA, when the secretory pathway is inactive. The simplest explanation is that peptide binding triggers a change in the conformation or dynamics of L^d^ that allows a tighter binding of B22.249 to HβP than to Hβ, resulting in a higher signal. This peptide-induced increase is consistent with a prior report showing that B22.249 is sensitive to peptide occupancy, particularly to the presence of a proline at P2, while 30-5-7 is not (55). Thus, we conclude that peptide binds to STF1/L^d^, turning surface L^d^-Hβ into L^d^-HβP.

In contrast, peptide addition at the end of the experiment did not cause a significant reduction in 64-3-7 staining on either control (Figs. 2*B* and 0 min), nor in cells under temperature shift treatment (Fig. 2*B*, 15 min). These results suggest that cell surface 64-3-7-positive molecules do not bind peptides, in accordance with prior findings showing that 64-3-7 specifically recognizes a form of L^d^ that is refractory to peptide rebinding at the plasma membrane (13, 16). Taken together, our data suggest that when used for staining cells, 64-3-7 recognizes surface L^d^-fH, which are present in appreciable amounts at steady state at 25 °C and increase rapidly after temperature shift.

In the analogous experiment with HeLa/US6 cells, we found a significant decrease in W6/32 levels after temperature shift (Fig. 2*F*, 30 min, no peptide) and a corresponding increase in HC10 labeling (Fig. 2*G*, 30 min, no peptide). These results suggest that HC10-positive molecules are HLA-fH that arise from HLA-Hβ after β_2_m dissociation (14). Peptide addition at 4 °C at the end of the experiment did not cause a significant change in W6/32 (Fig. 2*F*, 30 min) nor HC10 levels (Fig. 2*G*, 30 min). This indicates that HC10-positive molecules do not bind peptides at the plasma membrane, and that HC10 recognizes HLA-fH, which are present at low amounts at steady state at 25 °C. Taken together, the HLA data suggest that, like for L^d^, HLA-fH are generated by Hβ→fH decay through β_2_m dissociation. This pathway of fH generation is consistent with previous observations (14).

### MHC class I free heavy chains arise at the plasma membrane after β_2_m dissociation

The conversion of Hβ into fH explains the rapid loss of conformed (β_2_m-associated) class I molecules (B22.249-, 30-5-7S-, W6/32-positive) and the increase in fH signal (64-3-7, HC10) observed during the BFA/temperature upshift experiments. However, similar results would be expected if, upon the 25 → 37 °C shift, very fast Hβ endocytosis and fH recycling were to occur simultaneously.

To distinguish between these possible mechanisms, we monitored Hβ and fH levels over time (BFA/temperature upshift experiment, Fig. 3*A*). Following the 25 → 37 °C temperature shift in the presence of BFA, surface L^d^-Hβ levels (B22.249 and 30-5-7S) dropped by 50% within the first 5 min (Fig. 3*B*), while surface levels of L^d^-fH (64-3-7) increased within this time frame, doubling their initial amount (Fig. 3*B*). Hβ endocytosis followed by fH recycling cannot explain these observations, since recycling of L^d^-fH was described as a slow process, only detectable at later times (5, 6) (and see below). Instead, the increase in fH kinetically mirrored the decrease in Hβ at early times during the experiment. Thus, our results support the model in which the fH arise from Hβ (through β_2_m dissociation) at the cell surface right after the temperature shift. At later time points, the Hβ signal was below detection levels, whereas the fH signal was still detectable and declined gradually (Fig. 3*B*).

Taken together, our results show that at early time points, fH emerge from the Hβ pool faster than they are endocytosed, leading to their accumulation at the cell surface.

To derive quantitative information from the data, we modeled the generation (Hβ→fH) and endocytosis of the fH *in silico* (Supp. Information 1), assuming a simple model governed by the half-time of β_2_m dissociation (r_1_) and the half-times of Hβ (r_2_) and fH endocytosis (r_3_) (Fig. S2*A*), which fitted the experimental data for B22.249, 30-5-7S and 64-3-7 very well (Fig. 3*B*, solid line: B22.249 and 30-5-7S; short-dashed curve: 64-3-7). The β_2_m dissociation half-time (r_1_) from L^d^-Hβ was just 3 min, whereas the half-time of fH endocytosis (r_3_) was 90 min (Fig. S2*A*). In contrast, the endocytosis of Hβ (r_2_) is expected to be slower than r_1_, and therefore, for modeling purposes, we assigned a value of 100 min to r_2_ (Supp. Information 1). This assumption is consistent with the observation that fH surface accumulation mirrors the rapid loss of Hβ, which would not occur if Hβ were preferentially internalized rather than converted to fH at the surface.

The 28-14-8S mAb binds to the L^d^ α_3_ domain in a β_2_m-independent manner (13, 15, 51, 52, 53, 55) and should therefore bind to all L^d^ forms (L^d^-HβP, L^d^-Hβ and L^d^-fH). Accordingly, the 28-14-8S signal is expected to be the mathematical average of the 64-3-7 and 30-5-7S/B22.249 curves, weighted by their relative abundances before normalization. Interestingly, the predicted decay rate for 28-14-8S-positive L^d^ molecules (Fig. 3*B*, long-dashed curve) did not match the experimental data (Fig. 3*B*, triangles). This observation does not contradict the reliability of the model, since it accurately predicted the 28-14-8S signal decay in STF1/D^b^ cells (Fig. S2*B*, long-dashed curve). The α_3_ domains of L^d^ and D^b^ are identical, and thus, differential binding modes are unlikely to explain the observed differences. Rather, in L^d^-fH, the 28-14-8S epitope may be partially obscured by an unknown binding partner, leading to artificially low readouts.

In HeLa/US6 cells, likewise, the levels of β_2_m-associated HLA (W6/32) decreased rapidly upon the 25 → 37 °C temperature shift. An approximate 50% decrease of W6/32-positive molecules was detected within the first 30 min (Fig. 3*C*). At later time points, the W6/32 signal stabilized at an average of 26% of the initial value, perhaps due to an incomplete TAP inhibition by US6. Unlike genetic TAP deficiency, US6 acts as a viral inhibitor that blocks TAP function by direct interaction with the TAP complex and may allow residual peptide translocation and class I peptide loading. Additionally, HeLa-endogenous HLA allotypes might be partially TAP-independent, binding to ER-derived peptides. Therefore, at time zero, surface W6/32-positive molecules consist of ∼74% HLA-Hβ and ∼26% HLA-HβP, with the latter population being more stable and responsible for the sustained signal at later time points. This means that after 30 min of temperature shift, the remaining 47% of the W6/32 signal is composed of ∼21% HLA-Hβ and ∼26% HLA-HβP (Fig. 3, *C* and *D*), assuming equal binding of W6/32 to both species. Taking this into account, during the first 10 min of incubation at 37 °C, half of the initial pool of HLA-Hβ has undergone β_2_m dissociation (Fig. 3, *C* and *D*). This assumption is reproduced by the *in silico* model (Figs. 3*C* and S2*A*), with an r_1_ value of 10 min that fits the experimental data.

Mirroring this decrease in HLA-Hβ, the surface levels of HLA-fH (HC10) increased at early time points (Fig. 3*C*). This confirms that HLA-fH arise from HLA-Hβ (through β_2_m dissociation) at the cell surface right after the temperature shift. The initial increase in HC10 signal was substantial compared to the very low baseline levels at time zero (Fig. S1*C*). From 60 min after temperature shift, the signal for HLA-fH decayed at an almost constant rate (Fig. 3*C*). *In silico* modeling of the experimental data indicates that, once generated, the HLA-fH, and likewise murine fH, are slowly removed from the cell surface with a half-time of ∼145 min at 37 °C (r_3_, Fig. S2*A*).

Taken together, for HLA, like for L^d^, our results suggest that fH emerge from the Hβ pool by β_2_m dissociation following a 25 → 37 °C temperature shift, and that endocytosis of these newly generated fH is slower than their production rate, leading to their accumulation at the cell surface. While the *in silico* model reflects our experimental data well, the inferred proportion of HLA conformers should be interpreted with caution in the absence of an independent quantitative method to directly determine Hβ and HβP.

### Peptide binding to surface Hβ reveals a recycling pool of free heavy chains with different kinetics between the analyzed human and murine MHC class I allotypes

So far, our results suggest that fH arises after β_2_m dissociation from Hβ at the plasma membrane (PM) (Hβ^PM^→fH^PM^). We therefore asked whether fH are also generated in recycling endosomes (RE) (or similar compartments; Hβ^RE^→fH^RE^) followed by recycling of these fH to the cell surface (Hβ^RE^→fH^RE^→fH^PM^). We reasoned that this might be shown by blocking the first process (Hβ^PM^→fH^PM^) by having an exogenous peptide present at all times (Fig. 4*A*). Now, any increase in fH^PM^ over time would be due to fH^RE^ recycling from inside the cell.

As anticipated, constant peptide addition prevented the decrease in B22.249 and 28-14-8S signals (Fig. 4*B*), that normally occurs upon 25 → 37 °C temperature shift in the absence of peptide (Fig. 3*B*). Furthermore, no rapid increase in the 64-3-7 signal was observed early in the experiment, indicating that the increase in L^d^-fH observed in Fig. 3*B* is therefore not the result of a fast recycling of intracellular fH. Interestingly, 2 hours after the temperature shift, the surface fH levels remained constant. Likewise, no early steep increase in fH^PM^ was observed for D^b^-fH (Fig. S3*A*). Thus, at short times, β_2_m dissociation at the cell surface is the main source of murine fH^PM^, but on a longer time scale, slow fH^RE^ recycling from intracellular compartments might contribute to surface fH^PM^ levels. These findings agree with previous reports showing that the L^d^-fH recycling pool is associated with membranes of the late but not of the early endosomal compartment (5, 6).

In HeLa/US6, addition of peptides preserved the W6/32 signal during the first hour after temperature shift (Fig. 4*C*). However, unlike for the murine allotypes, HLA-fH still emerged at the surface despite peptide treatment (Fig. 4*C*), albeit at reduced levels (approximately one-third compared to untreated cells, Fig. 3*C*). This suggests that β_2_m dissociation at the cell surface is also the main source of HLA-fH^PM^ (Fig. 4*C*). Yet, some HLA-fH^RE^ also recycle from intracellular compartments, contributing to surface fH^PM^ levels in the time range from 1 to 3 hours.

### Peptide stabilization of surface Hβ reveals a substantial contribution of recycling endosomal Hβ to the formation of fH

In the constant presence of peptide, B22.249 and 28-14-8S signals decreased only slowly over time, demonstrating the long surface persistence of HβP complexes. Interestingly, B22.249 and 28-14-8S showed a slow increase at early time points (Fig. 4*B*). Since the anterograde secretory pathway is blocked by BFA, this observation suggests the existence of an intracellular pool of Hβ, possibly in recycling endosomes (Hβ^RE^), that moves to the cell surface. To understand the quantitative role of the Hβ^RE^ pool in the generation of fH^PM^, we modified the experiment by incubating the cells with peptide only before the 25 → 37 °C temperature shift, but not afterwards (Fig. 5*A*). We reasoned that any pre-existing Hβ^PM^ would thus be blocked from losing β_2_m, and Hβ^RE^ would decay to yield fH, since they would remain inaccessible to extracellular peptides (as suggested by results in Fig. 1, *B*–*D*). If this decay were to occur in the cell interior, then the resulting fH^RE^ does not rapidly travel to the surface (Fig. 4*B*); therefore, any rapid fH^PM^ increase would show Hβ^RE^→Hβ^PM^ recycling followed by Hβ^PM^→fH^PM^ decay.

At short incubation times, there was a rapid increase in the levels of L^d^-fH^PM^ (Fig. 5*B*) and D^b^-fH^PM^ (Fig. S3*B*), indicating that an intracellular Hβ pool is protected from binding exogenous peptide, and that upon temperature shift, these molecules rapidly recycle to the cell surface, and there, they decay into fH (Hβ^RE^→Hβ^PM^→fH^PM^). Under these conditions, the amount of fH formation (Figs. 5*B* and S3*B*) was lower than in the complete absence of exogenous peptides (Fig. 3*B* and S2*B*), but still substantial. The highest relative increase (peak) in 64-3-7 staining dropped from 2.0 (Fig. 3*B*) to 1.5 relative units (Fig. 5*B*) for L^d^-fH and from 1.55 (Fig. S2*B*) to 1.27 relative units (Fig. S3*C*) for D^b^-fH. This suggests that for both L^d^ and D^b^, one-third of the total Hβ^PM^ pool had been stabilized by peptide at the surface to give HβP^PM^, while two-thirds were recycled to the surface in a peptide-free state (Hβ^RE^→Hβ^PM^) and converted to fH^PM^ at 37 °C.

Taken together, for the studied murine MHC class I allotypes, we have shown that after overnight incubation at 25 °C, two populations of Hβ exist, Hβ^PM^ and Hβ^RE^. Upon a shift to 37 °C, Hβ^PM^ rapidly loses β_2_m, whereas Hβ^RE^ first moves to the cell surface and only then the dissociation of β_2_m occurs, being the main contributor to the fast generation of mouse fH^PM^. Recycling of fH^RE^ to the cell surface, in contrast, was not observed at early time points. The freshly generated fH are stable at the cell surface for hours.

In HeLa/US6, there was a strong (50%) decrease of W6/32-positive molecules within 30 min. After 2 hours, the W6/32 levels stabilized at an average of 23% of the initial value, perhaps due to incomplete TAP inhibition by US6 (Fig. 5*C*). This drop in W6/32 signal mirrored the kinetics observed in the absence of peptide (Fig. 3*C*). Likewise, total fH accumulation (Fig. 5*C*) was comparable to that observed when the peptide was omitted (Fig. 3*C*).

Taken together, we have shown that after overnight incubation at 25 °C, two populations of Hβ, Hβ^PM^ and Hβ^RE^, also exist in HeLa cells. Upon temperature shift to 37 °C, the contribution of Hβ^PM^ in the fast generation of fH is minimal. Instead, after moving to the cell surface, Hβ^RE^ lose β_2_m and give raise to most of the HLA-fH detected at the plasma membrane of HeLa/US6 cells. Recycling of HLA-fH^RE^ was also detected at short incubation times. The freshly generated fH are stable at the cell surface for hours.

## Discussion

While the presence of MHC class I free heavy chains (fH) at the cell surface is well documented (13, 14, 15, 16, 17, 18, 19, 20, 21, 22, 23, 24, 25, 26, 27, 28, 29, 30, 31, 32, 33, 34, 35, 36, 37, 38, 39), the mechanisms underlying their generation remain debated (8, 13, 14, 22, 23, 25, 26, 27, 28, 40, 41, 42). Surface fH (fH^PM^) was proposed to arise by β_2_m dissociation within endosomes, followed by recycling, or by dissociation directly at the plasma membrane after loss of peptide and β_2_m. Notably, fH^PM^ has been detected for hours after its initial appearance (13, 14), indicating relative stability once formed. However, the short-term dynamics of fH^PM^ emergence, particularly within minutes of surface localization, remained poorly defined.

**Figure 6:**
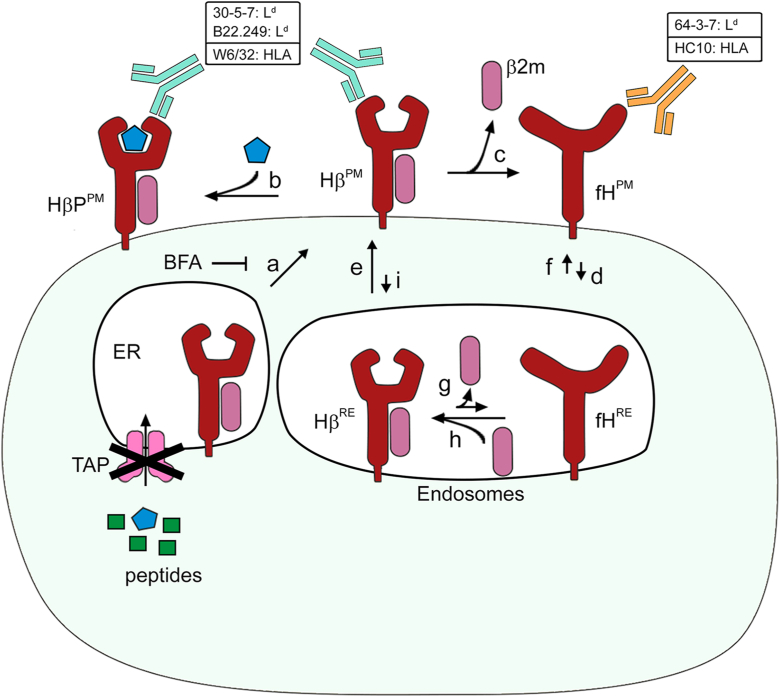
**Schematic representation of MHC class I species and the cellular and molecular processes governing their surface expression and turnover in TAP-deficient and TAP-inhibited cells under defined experimental conditions.** Peptide-free class I molecules (Hβ) accumulate at the plasma membrane at 25 °C but not at 37 °C (*arrow a*). Brefeldin A (BFA) inhibits anterograde transport from the endoplasmic reticulum (ER) to the cell surface but does not interfere with endocytosis, recycling or lysosomal targeting. Free heavy chains (fH) emerge at the plasma membrane after β_2_m dissociation at 37 °C (*arrow c*), unless exogenous peptides stabilize the Hβ complex (*arrow b*). The endocytic removal of Hβ during the experiment time scale is minimal (*arrow i*). The resulting fH accumulate at the surface due to a low endocytic rate (*arrow d*). Hβ recycling happens within minutes after the 25 → 37 °C temperature shift (*arrow e*). The recycled population is also peptide-receptive (*arrow b*) and in the absence of peptide contributes to fH formation (dissociation of β_2_m). The recycling of fH occurs at later time points for murine class I, but it can happen within minutes for HLA-fH (*arrow f*). Dissociation of β_2_m within endosomes is restricted for the murine Hβ L^d^ and D^b^ but occurs to a limited extent for some or all the endogenous HLA-Hβ expressed in HeLa cells (*arrow g*). Reassociation of β_2_m in recycling endosomes (or similar compartments) seems to occur for the murine allotypes L^d^ and D^b^ when expressed in STF1 (human cells) and to a limited extent for at least some of the endogenous HLA molecules expressed in HeLa/US6 cells (*arrow h*). Antibodies names are shown with the corresponding recognized allotype (*e.g.*, 30-5-7: L^d^). When a monoclonal antibody binds to more than one class I form (*e.g.*, both HβP and Hβ), this is illustrated by showing the same antibody pointing at each of the relevant forms. The schematic is not intended to represent precise binding epitopes, but rather to guide the reader in identifying which class I forms are recognized by each mAb.

To study fH^PM^, we used TAP-deficient and TAP-inhibited cells, which show very low surface levels of class I molecules loaded with endogenous peptides but accumulate “peptide-free” class I (Hβ^PM^) when incubated overnight at 20 to 28 °C (Fig. 6, arrow a). These molecules can bind exogenous peptides (Fig. 6, arrow b), which prevents β_2_m dissociation. When cells were instead incubated at 37 °C in the absence of peptide, we consistently observed a reciprocal shift in antibody reactivity: binding of all conformational antibodies that report on folded β_2_m-associated complexes (B22.249, 30-5-7, W6/32, Y3) decreased, while fH-specific antibodies (HC10, 64-3-7) increased. One possible explanation is that Hβ molecules undergo conformational rearrangements, partially unfolding to expose fH epitopes while still preserving β_2_m. However, this model is difficult to reconcile with the well-established observation that, in TAP-deficient cells, peptide-free class I molecules rapidly lose their association with β_2_m at physiological temperature (46). Thus, the easiest explanation is that β_2_m dissociation occurs after the 25 → 37 °C temperature shift, directly generating fH (Fig. 6, arrow c).

Consistent with previous studies (13, 14), we find that fH persists at the plasma membrane for hours after formation. Interestingly, their half-lives differ between class I allotypes, suggesting that fH^PM^ removal rates (Fig. 6, arrow d) depend on intrinsic properties of the fH or on differential interactions with clearance pathways. Notably, fH engage in homotypic and heterotypic interactions (43). Homotypic fH clustering appears to be allotype-dependent (our unpublished observations), and it is plausible that these interactions may influence their clearance rate from the cell surface. Our results with mAb 28-14-8S agree with this hypothesis. It recognizes an epitope in the α_3_ domain of L^d^ and D^b^ heavy chains, opposite to the β_2_m binding site, and is β_2_m-insensitive (51). Therefore, 28-14-8S should recognize all L^d^ and D^b^ forms, and its relative signal should reflect a weighted combination of the signals observed for B22.249 (detecting Hβ) and 64-3-7 (detecting fH), proportional to their relative abundance. Our *in silico* modelling of 28-14-8S reactivity closely matched the experimental data for D^b^ (Fig. S2*B*), but for L^d^, the reactivity was much less than expected (Fig. 3*B*). Since L^d^ and D^b^ α_3_ domains are 100% identical, the discrepancy is unlikely to be due to differential antibody binding but more plausibly reflects impaired recognition of L^d^-fH by sterical hindrance. This suggests that L^d^-fH participate in homotypic and/or heterotypic protein-protein interactions that mask the 28-14-8S epitope.

We have recently shown that K^b^-fH engage in homotypic interactions at the cell surface (10). This prompted us to test whether the 28-14-8S recognition of a K^b^ chimera carrying the D^b^/L^d^ α_3_ domain was similarly impaired. The 28-14-8S signal declined faster than predicted for this construct (Fig. S2*C*), together with an extended surface half-time of the resulting fH (∼200 min, nearly twice that of L^d^-fH and D^b^-fH (Fig. S2*A*, r_3_ value). These findings suggest that epitope masking correlates with engagement in homotypic interactions and prolonged surface retention. If confirmed, this would suggest that L^d^ and D^b^ are less prone to form such homotypic interactions compared to K^b^. Alternatively, heterotypic interactions involving K^b^-fH or intrinsic features unique to K^b^-fH may underlie the extended half-life. In conclusion, the accumulation of fH at the cell surface is likely due to slow internalization (Fig. 6, arrow d), potentially caused by, though not limited to, protein-protein interactions that retain them at the cell surface.

We also demonstrate that class I fH arise at the cell surface *via* β_2_m dissociation, both from resident Hβ molecules (Fig. 6, arrow c) and from recycling Hβ pools (Fig. 6, arrow e). Moreover, for the endogenous HLA allotypes expressed in HeLa cells, we detected an additional pool of fH that recycles from endosomes back to the cell surface upon shifting to 37 °C (Fig. 6, arrow f), although the contribution of individual allotypes cannot be resolved, as all carry the HC10 epitope. Whether this behavior extends to other allotypes remains unknown. In contrast, no rapid recycling was observed for L^d^-fH or D^b^-fH under comparable conditions. These findings point to potential differences in fH trafficking between human and murine MHC class I molecules that remain to be elucidated.

This observation suggests that murine L^d^-Hβ and D^b^-Hβ do not dissociate in intracellular recycling compartments (Fig. 6, arrow g), but only after reaching the cell surface (Fig. 6, arrows e and c). This may be explained by the high local concentration of free β_2_m in endosomal compartments, which may help stabilize Hβ *via* rapid re-association (Fig. 6, arrow h). Although the exact concentration of β_2_m in these compartments is not known, even a single β_2_m molecule in a 500 nm vesicle would correspond to ∼25 nM, a value above the reported β_2_m dissociation constants (0.2–0.5 nM *in vitro* (71); 1–10 nM on intact cells (72)). Structural studies showed that D^b^ binds more tightly to human β_2_m than to its murine counterpart (73). This may be also true for L^d^-Hβ, reducing its tendency to dissociate within endosomes and consistent with our observations. In contrast, HLA-Hβ may be only partially stabilized in endosomes, such that a fraction dissociates and the resulting fH can recycle back (Fig. 6, arrow f).

What is then the abundance of fH on the surface of a wild-type primary cell at steady state? Although published experiments have not provided direct quantification, we can use the measured half-times for an estimate. In the system

$$X\overset{r_{0}}{\to }{H\beta }^{PM}\overset{r_{1}}{\to }{fH}^{PM}\overset{r_{3}}{\to }X^{\prime }$$

where X denotes the source of the ${H\beta }^{PM}$ and X′ the endocytosed fH, and $r_{n}$ are the half-times shown in Fig. S2*A*, the ${fH}^{PM}$:${H\beta }^{PM}$ ratio at steady-state must equal the inverse ratio of their decay rate constants, *i.e.*, the ratio of their decay half-times (74), such that

$$\frac{c_{fH}}{c_{H\beta }}=\frac{r_{3}}{r_{1}}$$

Thus, for L^d^ at 37 °C and steady state, the fH^PM^:Hβ^PM^ ratio is ∼90:3, meaning that ∼30 fH^PM^ molecules are present for each Hβ^PM^. A similar calculation using the observation that an L^d^/peptide complex has a half-life of 7 h (75) suggests a rough estimate of 420:3 for the L^d^-HβP^PM^:L^d^-Hβ^PM^ ratio, and thus a relationship of 140:1:30 between HβP^PM^:Hβ^PM^:fH^PM^ for L^d^ at 37 °C and steady-state. There are 500 to over 3 × 10^6^ copies of MHC class I per cell, depending on the cell type and activation state (76). Assuming roughly 50,000 L^d^ molecules (measured as HβP) on the surface of a lymphocyte (77), this suggests about 11,000 L^d^-fH^PM^ at steady state. Numbers for other allotypes are likely to differ, since L^d^ has a comparatively low peptide binding affinity and surface abundance.

This calculation necessarily relies on a simplified, forward-directed scheme. In practice, some reactions are likely reversible, particularly within intracellular compartments where β_2_m concentrations are high and reassociation may occur. Nevertheless, under the experimental conditions tested, the net flux at the plasma membrane is dominated by β_2_m dissociation, making the forward approximation appropriate for describing surface fH dynamics.

Class I fH^PM^ were detected on primary human (14, 18, 19, 21, 24, 25, 27, 31, 39) and murine cells (14, 22, 26, 36), indicating that the presence of fH^PM^ is not restricted to cell lines. Consistent with this, primary murine splenic CD169^+^ macrophages, a myeloid population proficient in direct and indirect (cross-) antigen presentation *via* class I molecules, showed a significant increase in fH when cells were incubated at 25 °C, but not at 37 °C (Fig. S4*B*). Peptide addition at 25 °C prevented this increase. A similar trend was observed in splenic type 1 conventional dendritic cells, although statistical significance was not reached (not shown). This temperature-dependent fH^PM^ accumulation mirrors our observations in STF1/L^d^ (Fig. 1*E*) but is less pronounced in primary cells, likely due to their functional TAP transporter, which results in more efficient peptide loading. Likewise, B22.249 labeling in primary cells (Fig. S4*C*) showed a modest increase, suggesting that a subset of L^d^ molecules remains peptide receptive.

Interestingly, mildly acidic pH, as prevalent in endosomal compartments, can protect Hβ from dissociation *in vitro* and promote peptide exchange (78). Thus, the endosomal environment supports rather than destabilizes class I molecules. This is consistent with our observation of a recycling Hβ pool, and it supports a functional role for peptide loading or exchange in the endosomal pathway, in line with models of cross-presentation and intracellular antigen processing (79). Importantly, murine class I molecules exhibit increased stability and enhanced peptide exchange capacity when paired with human β_2_m (73). While this complicates direct interpretation, the detection of peptide-receptive class I at the surface of primary murine cells (Fig. S4*C*) indicates that these forms can arise under physiological conditions, suggesting they may be readily available for cross-presentation.

The sustained and measurable presence of fH at the plasma membrane, together with the expression of multiple immune receptors capable of recognizing them, points to regulatory functions (80). Early reports implicated fH in signal transduction and immune cell activation (18, 22, 24). In addition, soluble CD8α has been shown to bind fH (41), and CD8α and CD8β were reported to bind to “empty” L^d^ molecules (81). Building on this, our results raise the possibility that fH could act as sinks for soluble CD8, reducing its competition with membrane-bound CD8 on T-cells for peptide-MHC and co-receptor engagement during T cell activation.

Killer-cell immunoglobulin-like receptors (KIR) and leukocyte immunoglobulin-like receptors (LILR) play central roles in immune homeostasis but are also implicated in immune dysregulation (82). Several KIR/LILR-ligand interactions have been identified for HLA-HβP (83, 84, 85), for HLA-fH (86, 87), and for the disulfide-linked dimers of B27-fH (88, 89, 90). We have recently described non-covalent fH associations (10, 43). Class I fH clustering has been proposed to enhance antigen presentation by stabilizing the immunological synapse (38) and modulating the lateral mobility and rotational dynamics of HβP (91). Together, these findings suggest that fH may contribute to immune surveillance, particularly under conditions of impaired peptide loading, such as in some viral infections. The experimental tools we present here provide a means to test whether non-covalent fH clusters engage with KIR and LILR receptors, with implications for homeostatic control and immune dysfunction.

## Experimental procedures

### Plasmids

The mouse MHC class I genes were codon optimized for expression in mammalian cells, synthesized, and subcloned by GeneCust (Boynes, France) with appropriate flanking restriction sites. The gene products were subcloned into XhoI/XbaI or Xho/BamHI sites of the lentiviral transfer vector puc2CL6IEGwo, which contains an IRES-GFP cassette allowing the coexpression of the gene of interest and soluble GFP (92). DNA was resuspended in nuclease-free water and used for the transformation of competent *E. coli* DH5α cells. The lentiviral transfer plasmids pCD/NL-BH∗DDD (93) and pCMV-VSV-G (94) were obtained from Addgene (# 17531 and #8454, respectively). The human cytomegalovirus immunoevasin US6 bearing an HA-tag on its N-terminal domain was cloned into BamHI/XhoI sites of the lentiviral vector puc2CL6IPwo, which contains an IRES-PuroR cassette allowing the coexpression of the gene of interest and puromycin N-acetyltransferase for cell selection under puromycin treatment. Plasmid DNA was isolated using the ZymoPURE II Plasmid Midiprep kit (Zymo Research, Freiburg, Germany). H-2L^d^ was expressed in its wild-type form. The 64-3-7 epitope was transplanted into H-2D^b^ and H-2K^b^ through site-directed mutagenesis (R48Q in H-2D^b^ and R48Q, R50P in H-2K^b^), as previously defined (95). The α_3_ domain in H-2K^b^ was also replaced by the H-2L^d^/H-2D^b^ α_3_ domain. In the generation of this chimeric construct, amino acids 184 to 280 of the mature K^b^ molecule were replaced by the corresponding region from the mature L^d^, which is identical in D^b^ (residues 184–280 of the mature protein). This swap maintains the α_1/2_ domain of K^b^ while introducing the α_3_ domain from D^b^/L^d^.

### Cell lines and cell culture

STF1 cells were kindly provided by Henri de la Salle, (Etablissement de Transfusion Sanguine de Strasbourg, Strasbourg, France). HEK293T and HeLa cells were obtained from the American Type Culture Collection (ATCC). HeLa cells HLA^−/−^ were previously established (96) and were a kind gift from Prof. Anne Halenius, Universitätsklinikum Freiburg, Germany. Cell lines were routinely tested for *mycoplasma* contamination and confirmed to be free of *mycoplasma* throughout the course of the experiments. The hybridoma cell line secreting 64-3-7 antibody was a kind gift from Prof. Pero Lučin, University of Rijeka, Croatia. These cell lines were grown and maintained in High Glucose (4.5 g/L) Dulbecco's Modified Eagle Medium (DMEM) (Capricorn Scientific) supplemented with 2 mM L-glutamine (Capricorn Scientific), 100 U/ml streptomycin (Capricorn Scientific), 100 U/ml penicillin (Lonza) and 10% Fetal Bovine Serum (FBS, Capricorn Scientific) (Complete DMEM), at 37 °C in a 5% CO_2_ atmosphere. For routine passaging, cells at ∼80 to 90% confluency were washed once with DMEM and incubated with 0.05% trypsin-EDTA (Capricorn Scientific) at 37 °C until cell detachment was observed under a microscope. Then, cells were centrifuged after trypsin neutralization by the addition of an equal amount of complete growth medium. Cells were gently resuspended in complete growth medium and transferred to a new culture plate. Hybridoma cell lines producing the monoclonal antibodies B22.249, 28-14-8S, and HC10 were kind gifts from Prof. Alain Townsend, Oxford University, UK. W6/32 and 30-5-7S hybridomas were kind gifts from Prof. Anne Halenius, Universitätsklinikum Freiburg, Germany. These cells were grown in RPMI supplemented with 2 mM L-glutamine, 100 U/ml streptomycin, 100 U/ml penicillin, and 10% FBS. The hybridoma supernatants were collected, filtered through a 0.45 μm filter, supplemented with 0,01% NaN_3_, and stored at −20 °C until use.

### Antibodies, peptides, and reagents

The H-2L^d^ peptides RM9 (RPQASGVYM), YL9 (YPHFMPTNL), the HLA-A68 peptide GY9 (GQRKPATSY), the HLA-B15 peptide EA9 (ETSFVPSRA), the H-2D^b^ peptide FL9 (FAPGNYPAL), and the H-2K^b^ peptide SL8 (SIINFEKL) were from GeneCust (Boynes, France). All peptide stocks were prepared at 1 mg/ml in nuclease-free water from Promega (Walldorf, Germany) and stored at −80 °C before use. Allophycocyanin-conjugated goat anti-mouse IgG secondary antibody was from Jackson Immuno Research.

The following antibodies were used to identify splenic myeloid cell subsets by flow cytometry: TruStain FcX PLUS (anti-mouse CD16/32) Antibody (Biolegend, Cat# 156604, RRID:AB_2783138), anti-mouse CD11b, Phycoerythrin-Cy7 Conjugated (Rat, clone M1/70), Monoclonal Antibody (BD Biosciences Cat# 552850, RRID:AB_394491), anti-mouse CD11c (Hamster, clone N418) Brilliant Violet 711 Conjugated, Monoclonal antibody (Cat# 117329, RRID:AB_10897814), anti-mouse CD19 antibody (Rat, clone 6D5), Brilliant Violet 785 Conjugated, Monoclonal Antibody (Biolegend, Cat# 115543, RRID:AB_11218994), anti-mouse CD45 (Rat, clone 30-F11) Brilliant Violet 421 Conjugated, Monoclonal antibody (Biolegend, Cat# 103134, RRID:AB_2562559), anti-mouse CD64/FcgammaRI (Mouse, clone X54-5/7.1), PerCP/Cyanine5.5 Conjugated Monoclonal antibody (Biolegend, Cat# 139308, RRID:AB_2561963), anti-mouse CD169/Siglec-1, Phycoerythrin conjugated (Rat, clone 3D6.112), Monoclonal antibody (Biolegend, Cat# 142404, RRID: AB_10915697), anti-mouse F4/80 (Rat, clone BM8), Brilliant Violet 605 Conjugated, Monoclonal antibody (Biolegend, Cat# 123133, RRID:AB_2562305, anti-mouse I-A/I-E (Rat, clone M5/114.15.2), Alexa Fluor 700 conjugated, Monoclonal antibody, anti-mouse TCR beta chain (Rat, clone H57-597), Brilliant Violet 510 Conjugated, Monoclonal antibody (Biolegend Cat# 109234, RRID:AB_256235), anti-mouse/rat XCR1 antibody, (Mouse, clone ZET) Brilliant Violet 650 Conjugated, Monoclonal antibody (Biolegend, Cat# 148220, RRID:AB_2566410). Brefeldin A (cat. J62340.MF) was from ThermoFisher. Dimethyl Sulfoxide used in the preparation of BFA stock solutions was from AppliChem GmbH. OptiPrep, Axis Shield PoC AS was from Proteogenix (Cat# 1114542). Liberase DL (Dispase Low) Research Grade (Cat# LIBDL-RO) and DNase I recombinant, grade I, from bovine pancreas, expressed in Pichia pastoris (Cat# 04536282001) were from Sigma-Aldrich. 7-AAD Viability Staining Solution was from Biolegend (Cat# 420404).

### Cell transduction

Expression of mouse class I and US6 genes was achieved by lentiviral transduction. HEK293T cells were used to produce infecting viruses. The day before transfection, 2.2 ×10^6^ cells were seeded in a 10 cm dish. The following day, cells were at 70% confluency. High Glucose DMEM without FBS or antibiotics (450 μl) plus 50 μg linear 25,000 Da polyethyleneimine (PEI) dissolved in water (Sigma-Aldrich, cat. 40872), were incubated for 5 min at room temperature. Meanwhile, 6 μg of pCD/NL-BH∗DDD, 6 μg of pCMV-VSV-G and 6 μg of US6 or class I plasmids were added to 500 μl of high-glucose DMEM without FBS or antibiotics. The PEI and plasmid solutions were mixed and incubated for 15 min at room temperature. The media of HEK293T cells was replaced by 9 ml of fresh complete DMEM (see cell lines and cell culture section), and the DNA and PEI mix was added. Cells were grown at 37 °C in a 5% CO_2_ atmosphere for 24 h. After that, the cell medium was replaced by 10 ml of fresh complete DMEM and grown for another 24 h. The supernatant containing the viral particles was filtered through a 0.45 μm filter and kept at −20 °C until use. The day before transduction, 1.0 × 10^6^ STF1 or HeLa cells were seeded in a 10 cm dish and grown as described before. After 24 h, the medium was removed and replaced by 3 ml of fresh complete DMEM plus 5 ml of the filtered supernatant containing the viruses, and cells were incubated for 24 h. Next, the supernatant was removed, cells were trypsinized and seeded into a T75 flask and grown for an additional 24 h. This step was repeated twice. Cells transduced with the US6 plasmid were seeded at 20% and selected for 48 h with 2 μg/ml of puromycin. This procedure was repeated twice. The transduction efficiency of the mouse MHC constructs was assessed by analyzing GFP expression using flow cytometry. The transduction efficiency was always higher than 50%.

### Brefeldin A/temperature upshift (25 → 37 °C) decays

A total of 4 × 10^5^ cells were grown in 6-well plates at 37 °C with 5% CO_2_ for 6 h and then, cells were grown overnight at 25 °C with 5% CO_2_. After that, the medium was supplemented with 10 μg/ml brefeldin A (BFA, ThermoFischer) and incubated at 25 °C for 15 min to inhibit the anterograde transport of newly synthesized proteins. Next, the medium was removed and replaced by 2 ml prewarmed complete DMEM supplemented with 10 μg/ml BFA and incubated at 37 °C for the indicated times. At the end of each time point, the cells were shortly trypsinized, harvested, and processed for flow cytometry. Cells that were only incubated at 25 °C were processed in parallel and used to normalize the results (time zero). When indicated, cell media or cell suspension was supplemented with 10 μM peptide.

### Primary cells experiments

Spleens were dissected from euthanized male or female 9 to 15 weeks-old Balb/c mice, perfused with 500 μg/ml Liberase DL Research grade (Sigma-Adrich) and 50 ng/ml recombinant DNase I (Sigma-Adrich) in RPMI, cut into 4 to 5 small pieces with scissors and digested for 30 to 40 min on a ThermoStat plus thermomixer (Eppendorf) at 800 rpm. Mechanical disruption was performed after 10 to 15 min of enzymatic digestion by passing the pieces 3 to 4 times through a 22G-needle attached to a 1 ml syringe. The digestion was stopped by putting the vials on ice and diluting cell suspensions in RPMI 10% FBS, 5 mM EDTA. After washing with RPMI supplemented with 10% FBS, Penicillin and Streptomyicn, suspension cells were incubated in 6-well plates with either in the absence or in the presence of RM9 peptide (50 μM) for 16-18 h at either 37 °C or 25 °C in a 5% CO2 atmosphere. Cells were then collected in 15 ml conical tubes and washed twice in PBS. Myeloid cells were enriched by performing a very-low-density Optiprep gradient centrifugation as described before (97), immunolabeled and processed for flow cytometry.

### Antibody staining and flow cytometry

Cells were shortly trypsinized, transferred to 1.5 ml tubes on ice containing 500 μl of DMEM + 10% FBS and centrifuged at 4 °C. Pellets were washed in 100 μl of cold PBS supplemented with 2% bovine serum albumin (PBS-BSA). After that, cells were resuspended in 100 μl of the corresponding hybridoma supernatant supplemented with 2% BSA and incubated on ice for 30 min. All staining was thus performed in medium containing β_2_m from fetal calf serum. After one wash in cold PBS-BSA, cells were resuspended in 50 μl of PBS-BSA containing 1/200 dilution of Allophycocyanin-conjugated goat anti-mouse IgG secondary antibody (Jackson ImmunoResearch Laboratories) and incubated for 30 min on ice. After a final wash in cold PBS, cells were resuspended in 1.5 ml cold PBS, and fluorescence data were collected using a Partec CyFlow Space cytometer (Sysmex Europe).

Primary cells were transferred to 96-well round-bottom plates and haversted by centrifugation at 4 °C. Pellets were washed in 200 μl of cold PBS twice. Cells were resuspended in 100 μl of the corresponding hybridoma supernatant supplemented with 2% FBS 2 mM EDTA, and incubated on ice for 30 min. After one wash in cold PBS, cells were resupended in 50 μl of PBS, FBS 2% EDTA 2 mM, containing 1/200 dilution of FITC-conjugated goat anti-mouse IgG secondary antibody and incubated for 15 min on ice. After two washes with PBS, cells were incubated with TruStain FcX PLUS antibody for 10 min before the addition of anti-CD11b, anti-CD11c, anti-CD19, anti-CD45, anti-CD64, anti-CD169, anti-F4/80, anti-MHC-II, anti-TCR-β, and anti-XCR1 antibodies, all diluted in PBS 2% FBS 2 mM EDTA for 30 min on ice. After two washes in cold PBS, cells were resuspended in PBS, 2% FBS 2 mM EDTA. 7-AAD (5 ml/ml) was added 5 to 10 min before data collection. Fluorescence data were recorded on a BD LSR Fortessa flow cytometer analyzer (BD Biosciences). Myeloid cell subsets were identified by adopting a gating strategy described elsewhere (97). The final analysis was conducted in FlowJo. Results were analyzed and tabulated in Microsoft Excel and the plots were prepared using GraphPad Prism. Final panels were compiled using Adobe Photoshop CS6.

### Statistical analysis

Statistical analyses were performed using GraphPad Prism 8 with a significance level of 0.05 and are explained in detail in the corresponding figure legends.

### *In silico* model

The reaction scheme in Fig. S2*A* was converted into a set of equations, assuming first-order reactions, and implemented in Microsoft Excel using the explicit Euler method (98), such that

$$c_{n}\left(t+\Delta t\right)=c_{n}\left(t\right)+\Delta t\cdot c_{n}^{\prime }\left(t\right)$$

with $c_{n}\left(t\right)$ being the concentration of n at time t, and $c_{n}^{\prime }\left(t\right)$ its change over time, such that

$$c_{n}^{\prime }\left(t\right)=\sum_{i}k_{i}\prod_{j}c_{ij}$$

with $k_{i}$ being the rate constant, calculated from the half-times $r_{i}$ according to

$$k_{n}=\frac{\ln\;2}{r_{n}}\text{,}$$

and *ij* being the participants of reaction i, yielding the following reaction rate equations:

$$c_{H\beta }^{\prime }\left(t\right)=-\left(k_{1}+k_{2}\right)\cdot c_{H\beta }$$

$$c_{fH}^{\prime }\left(t\right)=k_{1}\cdot c_{H\beta }-k_{3}\cdot c_{fH}$$

for the half-times $r_{1}$, $r_{2}$, and $r_{3}$, and the concentrations of Hβ and fH as shown in Fig. S2*A*. The step width of the simulation (Δt) was 1 s, and the duration was 364.5 min (21,870 steps). Absolute starting concentrations and reaction rates were fitted by hand, or by Microsoft Solver, to the laboratory data.

## Data availability

All data supporting the findings of this study are available within the manuscript and its supplementary information files. The raw datasets underlying the figures and analyses are available from the corresponding author upon request.

## Supporting information

This article contains supporting information.

## Conflict of interest

The authors declare that they do not have any conflicts of interest with the content of this article.
