# Supplementary material for: Surface emergence and persistence of MHC class I free heavy chains
Source: J Biol Chem. 2025 Oct 9;301(11):110799. doi: 10.1016/j.jbc.2025.110799 (PMC12630360; doi:10.1016/j.jbc.2025.110799)
Supplement: Supporting Figures [file mmc2.pdf]

Surface emergence and persistence of MHC class I free heavy chains

Fernando M. Ruggiero, François-Xavier Mauvais, Ursula Wellbrock, Peter M. van Endert and Sebastian Springer

This file contains supplementary figures 1-4

A

mAb	HβP	Hβ	fH	recognized allotype
28-14-8S	✓	✓	✓ *	H-2D^b, H-2L^d
30-5-7S	✓	✓	✗	H-2L^d
B22.249	✓	✓	✗	H-2D^b, H-2L^d
64-3-7	✗	✗	✓	H-2L^d, transplantation possible**
W6/32	✓	✓	✗	HLA-I
HC10	✗	✗	✓	HLA-I carrying ^{57}PxxWDR^{62}

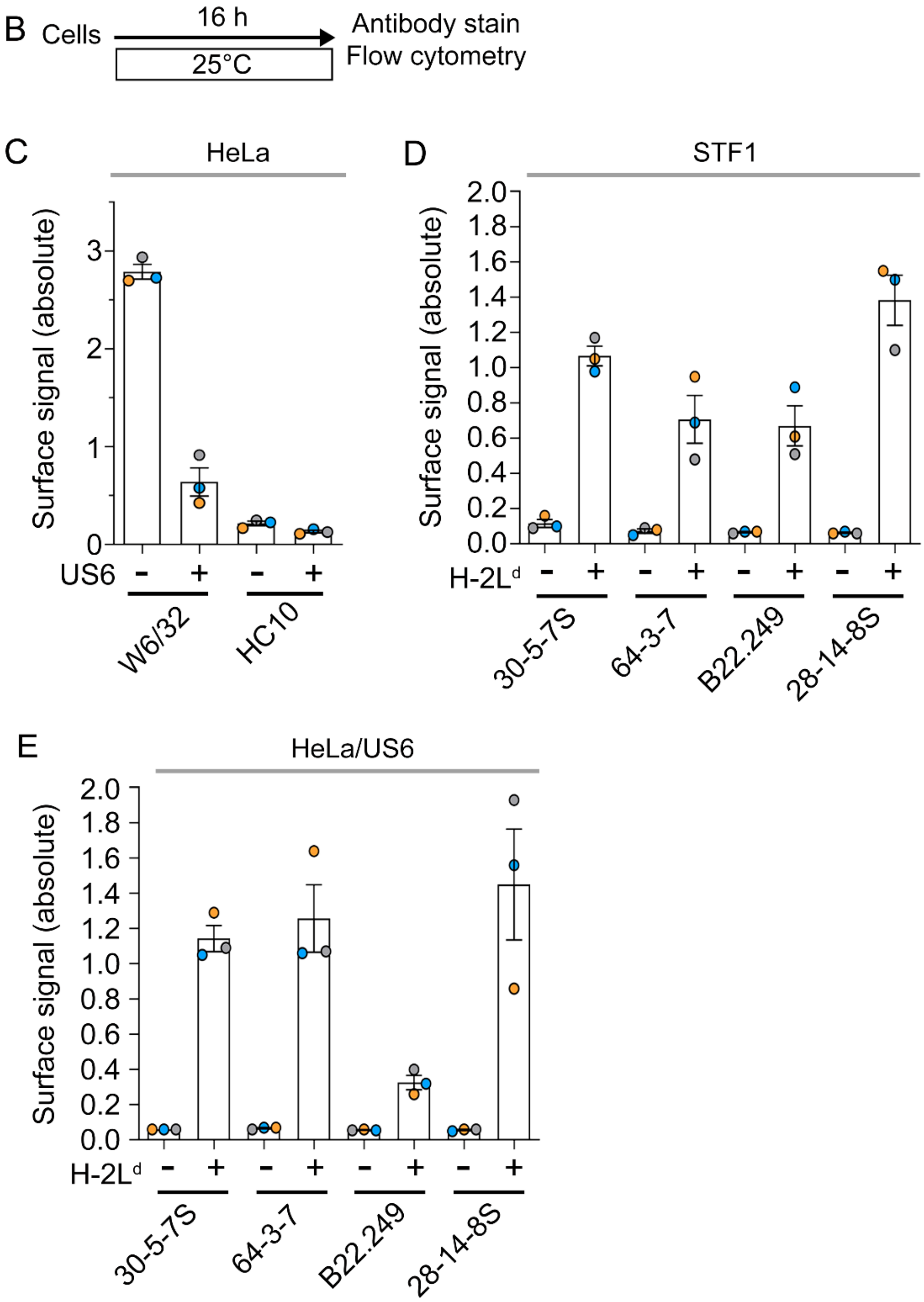

Supplementary Figure 1

A. Monoclonal antibodies (mAb) used in this study. The tick and cross marks indicate the class I species that the mAb can and cannot bind, respectively. * MAb 28-14-8S binds all forms of L^d and D^b, however, here we show that L^d-fH are not well recognized at the cell surface by this mAb. ** Limited mutagenesis allows the transfer of 64-3-7 epitope to other class I molecules.

B-E. Experimental procedure to accumulate surface class I molecules in HeLa wild type and HeLa/US6 (C), STF1 wild type and STF1/L^d (D) and HeLa/US6 ± L^d cells (E). Surface class I levels shown in panels C-E are the absolute mean fluorescence values obtained for each of the indicated conditions and mAbs. Each data point represents one independent biological experiment (mean of technical replicates). Three independent experiments per condition. Data are presented as mean ± S.E.M.

A

	STF1			HeLa/US6
	H-2L^d	H-2D^{b*}	H-2K^{b**}	endogenous
r_1	3	6	14	10
r_3	90	100	200	145
**fH (t=0)**	43	55	38	15

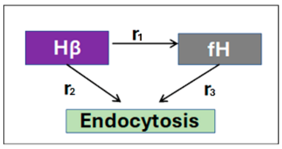

B

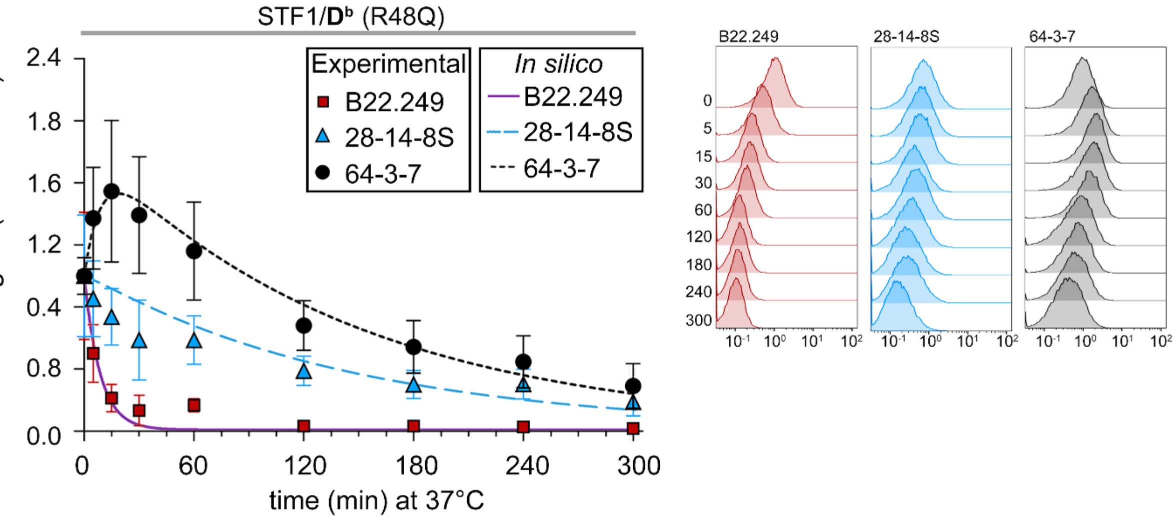

C

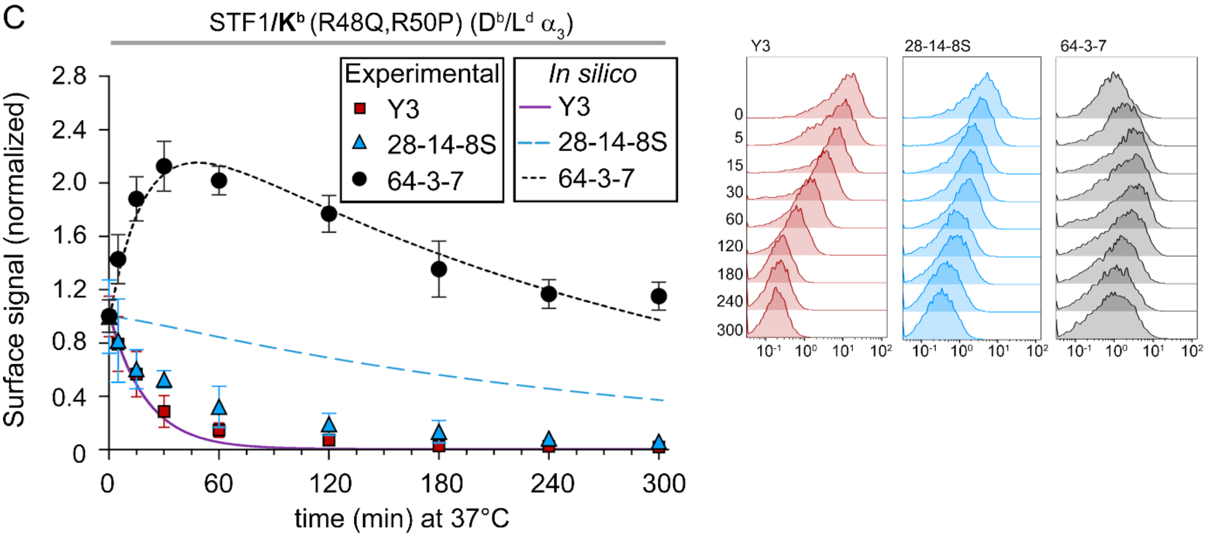

Supplementary Figure 2.

A. Integration of experimental data and *in silico* predictions to determine the β_2m dissociation (r_1)- and fH removal (r_3)-half-times for mouse and human class I molecules. These kinetic parameters were estimated based on the relative intensities and time-course of the signals obtained for Hβ- (B22.249 and W6/32) and fH-detecting mAbs (64-3-7 and HC10), and assuming, that the height of the fH peaks reflects the amount of Hβ that undergoes conversion to fH upon β_2m dissociation. Other consideration is that the Hβ removal by endocytosis (r_2) is significantly slower than r_1. The initial relative amount of fH on the cell surface at time zero (fH

t=0: [fH]/([fH]+[Hβ]) must be considered as well and is variable among class I allotypes/cell lines. */** Limited mutagenesis was used to introduce the 64-3-7 epitope in H-2D^b:(R48Q) and H-2K^b: (R48Q,R50P), the latter also engineered to carry the D^b/L^d α_3 domain, rendering it sensitive to recognition by mAb 28-14-8S.

B-C. Cells were incubated overnight at 25 °C, followed by 15 minutes at 25 °C in DMEM containing 10 µg/mL brefeldin A (BFA). The medium was then replaced by 37 °C pre warmed DMEM containing 10 µg/mL BFA, and cells were incubated at 37 °C for the indicated times. Surface class I levels were monitored by flow cytometry using the indicated mAbs in STF1/D^b (R48Q) (B) and STF1/K^b (R48Q,R50P) (D^b/L^d α_3) cells (C). An overview of the class I species recognized by each mAb is provided in Suppl. Fig. 1A. The plots show the fluorescence intensity for each mAb, at each time point, and relative to the corresponding control (0 minutes of incubation at 37 °C). Data are presented as mean ± S.E.M. *In silico* predictions are overlaid as follows: long-dashed curves for 28-14-8S (B-C); solid lines for B22.249 (B), and Y3 (C); short-dashed curves for 64-3-7 (B-C). At least three independent experiments were conducted. Representative flow cytometry histograms are shown next to each graph, with the x-axis showing the absolute fluorescence intensity values. The time that cells were incubated at 37 °C (minutes) is indicated to the left of the tracings.

A
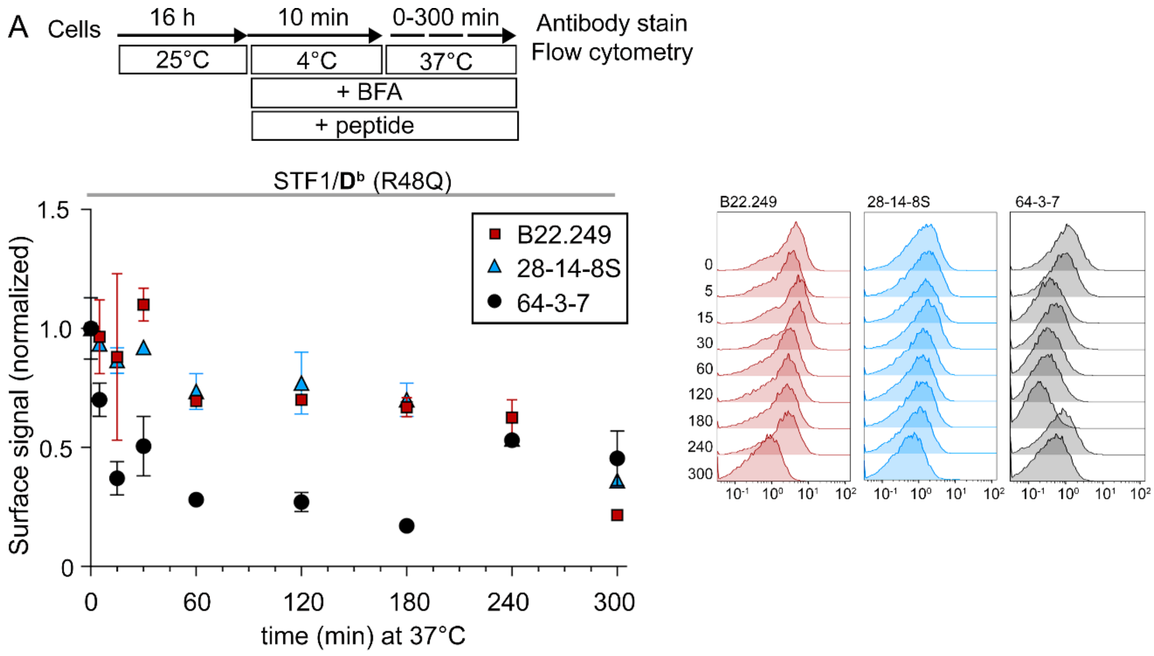

B
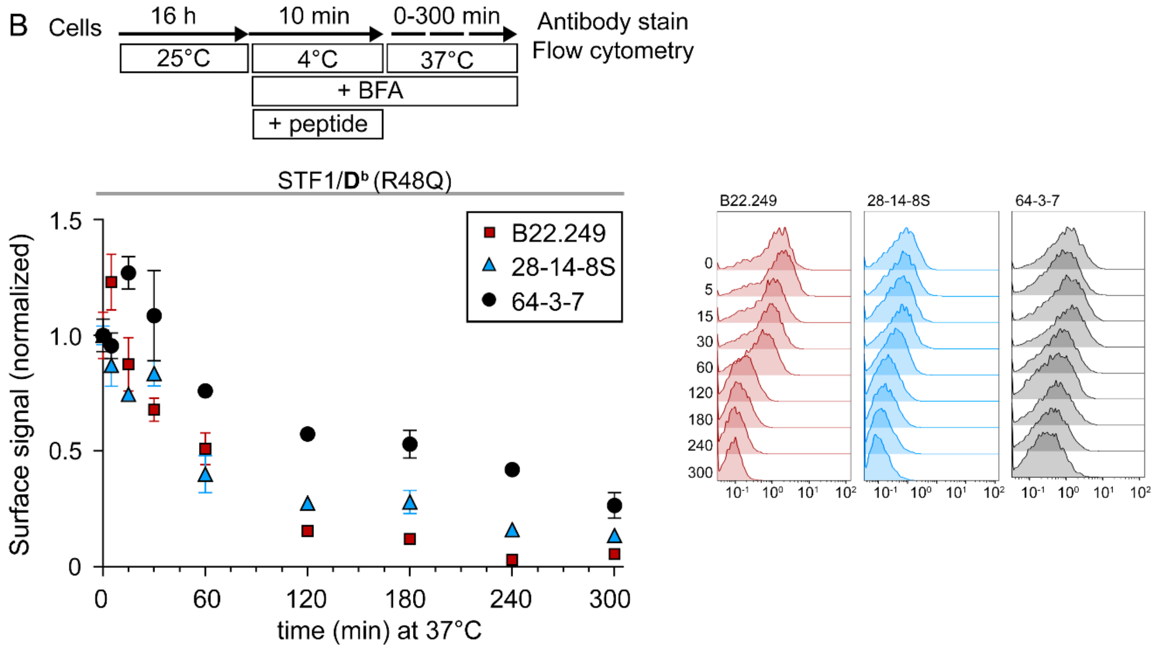

C

Cell line	STF1		HeLa/US6		
Allotype	H-2L^d	H-2D^b *	endogenous		
TAP	deficient	deficient	inhibited		
	2,00	1,55	5,55	no peptide (Fig. 3, Suppl. Fig. 2)	max fH detected (rel. to control)
	1,00	1,00	1,80	peptide at 4°C + 37°C (Fig. 4, Suppl. Fig. 3A)	
	1,50	1,27	6,70	peptide at 4°C (Fig. 5, Suppl Fig 3B)	
	≈ 1 : 2	≈ 1 : 2	mainly Hβ^{RE}	Hβ^{PM}/Hβ^{RE}	

Supplementary Figure 3

A. STF1/D^b cells were incubated overnight at 25 °C, followed by 10 minutes at 4 °C in DMEM containing 10 µM peptide and 10 µg/mL brefeldin A (BFA). The medium was then replaced with 37 °C pre-warmed DMEM containing 10 µM peptide and 10 µg/mL BFA, and cells were incubated at 37 °C for the indicated times. The specified mAbs were used to monitor the amounts of Hβ and fH at the cell surface. An overview of the class I species recognized by each mAb is provided in Suppl. Fig. 1A. The plots show the fluorescence intensity for each mAb, at each time point, and relative to the corresponding control (0 minutes of incubation at 37 °C). Data are presented as mean ± S.E.M. Limited mutagenesis was used to introduce the 64-3-7 epitope in H-2D^b (R48Q). Two independent experiments for each condition were conducted. Representative flow cytometry histograms are shown next to each graph, with the x-axis showing the absolute fluorescence intensity values. The time that cells were incubated at 37 °C (minutes) is indicated to the left of the tracings

B. STF1/D^b cells were incubated overnight at 25 °C, then transferred to 4 °C for 10 minutes in DMEM containing 10 µM peptide and 10 µg/mL brefeldin A (BFA). After washing, the medium was replaced with 37 °C pre-warmed DMEM containing 10 µg/mL BFA, and cells were incubated at 37 °C for the indicated times. D^b surface levels were measured by flow cytometry using the indicated monoclonal antibodies. An overview of the class I species recognized by each mAb is provided in Suppl. Fig. 1A. The plots show the fluorescence intensity for each mAb, at each time point, and relative to the corresponding control (0 minutes of incubation at 37 °C). Data are presented as mean ± S.E.M. Limited mutagenesis was used to introduce the 64-3-7 epitope in H-2D^b (R48Q). Two independent experiments for each condition were conducted. Representative flow cytometry histograms are shown next to each graph, with the x-axis showing the absolute fluorescence intensity values. The time that cells were incubated at 37 °C (minutes) is indicated to the left of the tracings.

C. Determination of the ratio between the Hβ at the plasma membrane ($H\beta^{PM}$) and Hβ at recycling endosomes ($H\beta^{RE}$) for the analyzed class I molecules.

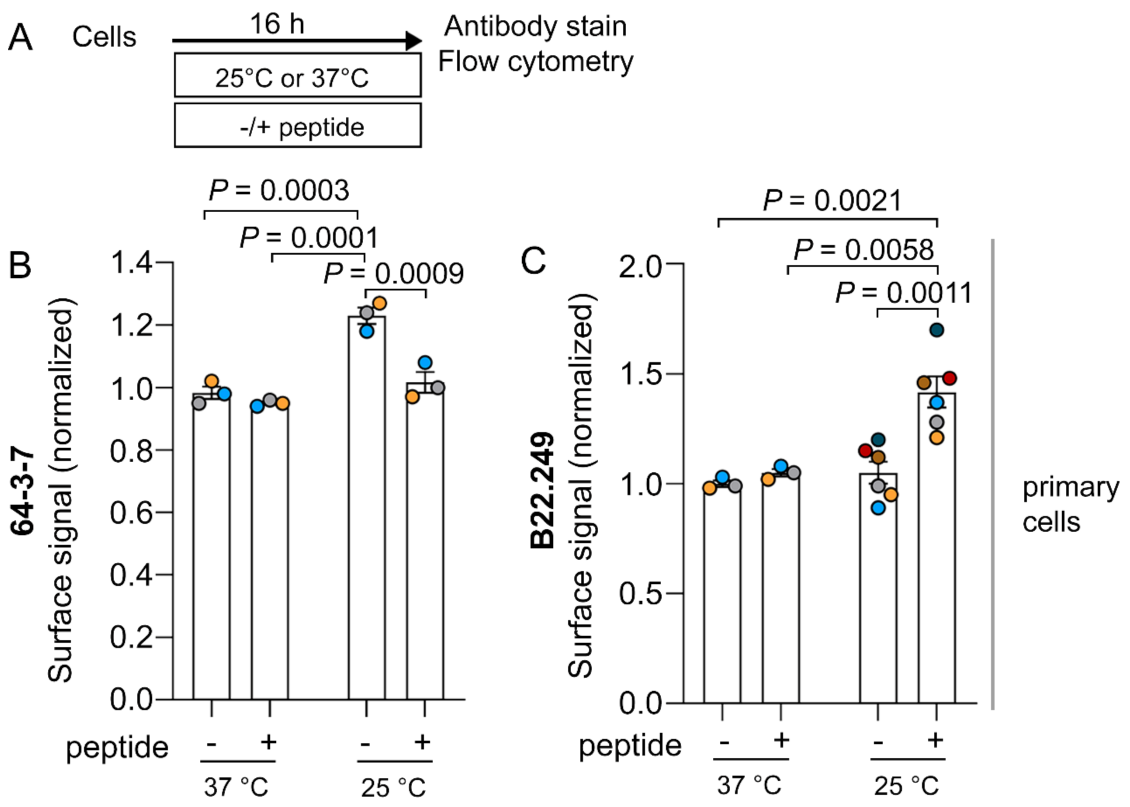

Supplementary Figure 4. Cell surface expression of class I-Hβ and -fH on murine primary cells

A. Primary splenic cells obtained from BALB/c mice were incubated overnight at 37 °C or 25 °C in the presence or absence of 50 μM peptides.

B-C. Cells were processed for flow cytometry using mAbs 64-3-7 (B) or B22.249 (C). Values are expressed as multiples of the signal obtained at 37 °C in the absence of peptides (control) for CD169^{+} splenic resident tissue macrophages, a myeloid cell subset capable of highly efficient MHC-I antigen cross-presentation. The presence (+) or absence (-) of peptide and the incubation temperature (37 or 25 °C) are indicated below. Statistical significance analysis was assessed using two-way ANOVA followed by Tukey´s multiple comparison test. Only significant changes are indicated with their corresponding *P* values. Between one and two independent experiments were performed. In each experiment, cells from three mice were processed independently. Data are presented as mean ± SEM, with each data point corresponding to cells from a single animal.
